# Do scarcity-related cues affect the sustained attentional performance of the poor and the rich differently?

**DOI:** 10.1098/rsos.251758

**Published:** 2025-11-26

**Authors:** Peter Szecsi, Miklos Bognar, Barnabas Szaszi

**Affiliations:** ^1^ Doctoral School of Psychology, ELTE Eötvös Loránd University, Budapest, Hungary; ^2^ Institute of Psychology, ELTE Eötvös Loránd University, Budapest, Hungary; ^3^ Corvinus Institute for Advanced Studies (CIAS), Corvinus University of Budapest, Budapest, Hungary

**Keywords:** poverty, cognition, policy, alleviation

## Abstract

Cues related to financial scarcity are commonly present in the daily environment shaping people’s mental lives. However, prior findings are mixed on whether such scarcity-related cues disproportionately deteriorate the cognitive performance of poorer versus richer individuals. In our registered report, we collected a large study sample (*N* = 4280) using targeted sampling strategies to reach a diverse group of people along education and financial status. We focused on attentional performance to—compared to prior studies—more sensitively assess the effect of even brief lapses of attention. Using words related to absolute scarcity (poverty) and relative scarcity (abundance) as cues, we found strong evidence against the existence of a different effect on the sustained attentional performance between poorer and richer participants. The utilized cues facilitated scarcity-related thoughts but not financial worries, which may explain the absence of the effect. The findings were robust across various analytical choices, including the used outcome variable, exclusion criteria, outlier treatment and used socioeconomic indicators. Our results suggest that, in online contexts, exposure to scarcity-related words does not differentially impact sustained attentional performance across socioeconomic groups, highlighting important boundaries to the generalizability of scarcity theory.

## 1. Introduction

Financial scarcity-related cues can create distinct mental experiences for individuals from different socioeconomic backgrounds [1–3]. While they may not affect the wealthy, they might lead to a decline in the cognitive performance of the poor [4]. Since scarcity-related cues are present in a multitude of daily experiences such as the clothing of a classmate (e.g. Rolex watch) or the language of a conversation (e.g. mention of utility bills), the negative impact can be pervasive and have lasting personal consequences. For instance, if being exposed to scarcity-related cues during a recruitment interview deteriorates the cognitive performance of low candidates disproportionately, it will increase societal inequities by making it harder for the poor to get hired even when being equally skilled compared to their middle- or high-income peers.

There is still no comprehensive theory that can explain when and for whom financial scarcity-related cues have a detrimental effect, although there is consensus among different theories on certain aspects of the phenomenon. Most theoretical accounts agree that financial worries of lower-income individuals lead to a decline in attentional performance, which in turn deteriorates higher-level cognitive performance as well^1^ [2,3,7,10]. Theories suggest that this effect is driven by two main factors: increased attentional tunnelling (i.e. increased attention on scarcity-related cues) and increased cognitive load (i.e. the mental capacity of poorer people decreases to a greater extent) [1,9]. While the increasing cognitive load harms performance in all tasks, tunnelling literature suggests that performance is improved in tasks where attention needs to be allocated to find scarcity-related cues, while it declines when the cues are unrelated to scarcity [11]. The impeding effect of scarcity-related cues on attentional performance is supported by recent findings showing that financial concerns [12] or general concerns about the future impede task performance [13,14], and out of the possible types of concerns, worries have the strongest detrimental effects on performance [15]. The term *Scarcity Salience Induced Cognitive Inequality* (SICI) will be used throughout this article to refer to the phenomenon whereby financial scarcity-related cues disproportionately affect the cognitive performance of the poor.

SICI has been observed both in laboratory studies and in a real-world setting. In one of the most cited psychological studies of the last decade, Mani *et al*. [2] increased the salience of financial scarcity by asking participants to find solutions for hypothetical financial problems if they were to happen to them. They found that increasing the severity of the hypothetical financial problem led to a larger decrease in the cognitive control and fluid intelligence performance of lower-income individuals, but not of higher-income participants. Duquennois [4] analysed three large educational datasets from various countries and found that when the ratio of questions related to money was higher in a test, students from schools in lower-income neighbourhoods performed worse. Although the conclusions in this study were based on indirect or macro level indicators used as proxies (school-level poverty or the educational level of the parents), this finding serves as valuable evidence about the potential real-life impact of SICI. While the existence of SICI has been supported also in an American [16] university student sample, other results questioned the generalizability of these studies showing that results may not hold across all settings and populations. Several studies failed to observe SICI in American [17–19], UK [20] and Hungarian [21,22] samples. Furthermore, Dang *et al*. [23] demonstrated on a Chinese student sample that poorer participants performed better in the presence of scarcity-related cues. In a recent systematic review of the SICI literature, screening 52 162 articles, Szecsi & Szaszi [24] identified 13 relevant experimental or quasi-experimental studies that examined the effect of financial scarcity-related stimuli on cognitive functioning. Their findings suggest that it is difficult to draw definitive conclusions about the existence of the SICI due to the highly heterogeneous findings (*g* = 0.07 [−0.02, 0.16], τ^2^ = 0.14 [0.07, 0.26])^2^.

The heterogeneity of prior findings raises the question of whether SICI systematically varies across different types of populations. For example, when exposed to scarcity-related cues, individuals experiencing objective absolute levels of poverty (the financial state of having insufficient resources to meet basic needs) [26] may have different reactions than individuals experiencing subjective relative (e.g. one’s perceived low financial status in comparison to their peers) or subjective absolute (e.g. the feeling of not having enough to get by) poverty. The way we operationalize these dimensions of poverty can also matter. While prior studies mainly focused solely on income, financial slack (i.e. available funds for unforeseen expenses) or indebtedness could also be relevant dimensions of objective absolute poverty. Owning some slack could reduce the pressure and financial worries that emerge with financial scarcity-related cues [27–29], while indebtedness could have the opposite effect [27,30]. Other factors can also affect SICI through their impact on the volume of stress related to personal finances: the lack of supportive social networks such as close family or friends [28], uncertainty about the timing and magnitude of one’s income [31,32], adverse childhood experiences caused by poverty experienced during childhood [33,34], having children or elderly relatives to care for, or not being financially dependent on others [28,35] can all play a role.

Although prior studies did not investigate the question, it is also possible that different types of scarcity-related cues have different effects on the cognitive performance of the poor. Prior empirical investigations predominantly used stimuli describing absolute scarcity: participants had to imagine how they would deal with different levels of financial shocks (e.g. [2]). However, cues of abundance (the sight of a luxury watch, a nice car or a big house) might also affect cognition [10,36,37]. That way, seeing the affluence of others (i.e. relative scarcity) can also remind individuals about their own deprivation. This can induce financial worries, and in turn, deteriorate cognitive performance.

In the present Registered Report, we conduct an experimental study to examine the moderating role of financial status on the impact of financial scarcity- and abundance-related cues on sustained attentional performance in online contexts (see table 1 for the overview of the hypothesis testing procedure). We aimed to make three contributions beyond the current state of the literature. First, we conducted a sufficiently powered experiment to identify even small effects, which is more in line with the effect sizes observed in prior findings. Prior experimental studies used a median sample size of 96 [24] enabling them to identify implausibly large effects (*d >* 0.74 with 0.95 power and 0.05 alpha^3^). Here, we aimed to collect a sample of at least 3000 individuals after exclusion criteria were applied that would let us detect an effect of 0.13 Cohen’s *d* with a statistical power of 0.80. This target sample size is larger than the sum of all observations in a recent systematic review [24].

**Table 1.** Design table.

question	hypothesis	sampling plan	analysis plan	rationale for deciding the sensitivity of the test for confirming or disconfirming the hypothesis	interpretation given different outcomes	theory that could be shown wrong by the outcomes
Does financial status moderate the impact of financial scarcity-related cues on sustained attentional performance in online contexts?	Financial status measured in Poverty Index score influences the difference in performance measured in Attentional Performance Index between the two experimental conditions, in a way that the higher the Poverty Index score, the worse participants perform in the Scarcity condition, while performance in the control condition is independent of the financial status of the participants.	We aim to reach 3000 responses after exclusion criteria are applied. We aim to collect at least 50 participants for each of the 24 different demographic groups determined by educational level and income. For details, see the *Power Analysis* section of the manuscript and the *Recruitment Group Descriptions* section of the electronic supplementary materials	We will compare linear regression models including and excluding the target regression term. For details, see the *Statistical Framework for Inferences* and *Main Analysis: SICI in the General Population* sections.	The sample size *N* = 3000 was selected based on our resources for data collection.	We will use p-values at the 0.05 significance level and equivalence testing to draw conclusions about the existence of the effect. IF the *p*-value of the interaction term is non-significant AND the equivalence test does not indicate an equivalence to zero, THEN we will conclude that we did not find evidence for the alternative hypothesis. ELSE IF the (p-value of the interaction term is non-significant AND the equivalence test indicates an equivalence to zero) OR the *p*-value of the interaction term is significant but in the non-predicted direction, THEN we will conclude that we did find evidence for the null hypothesis. ELSE IF the interaction term is significant in the predicted direction, THEN we will conclude that we found evidence in favour of the alternative hypothesis. For details, see the *Statistical Framework for Inferences* section.	Scarcity theory, partially. Specifically, the prediction that textual cues related to scarcity impede cognitive performance in general.

Second, we assessed the generalizability of SICI by exploring in which populations, and for what type of scarcity-related cues it arises. We used both convenience and targeted sampling strategies to reach a diverse range of the Hungarian population with respect to income, education and age, enabling us to examine the generalizability of SICI within the Hungarian context. Additionally, we measured numerous potential moderators of the effect (e.g. dimensions of experienced poverty, extent of social network). Furthermore, we experimentally manipulated whether the cues in the experimental condition are related to relative or absolute scarcity to investigate whether this characteristic affects the magnitude of the SICI.

Third, our experiment improves the prevalently used study design in the literature on several dimensions. First, in the control condition, the presented words and expressions were semantically unrelated to relative or absolute scarcity, while in the scarcity salience condition, participants would encounter words and expressions related to financial scarcity. That enables us to test whether the mere presence of financial scarcity-related cues affects the cognitive performance of the poor disproportionately. Second, as part of the experimental manipulation, participants were asked to generate free associations related to the presented words. This manipulation is intended to mimic the existing processes in real-world situations (e.g. distracting thoughts coming to mind when encountering an exam question with financial words) and enable us to explore how people’s cognition is affected when they face such cues in their everyday lives. Finally, we measured participants’ cognitive performance using a sustained attention task (Sustained Attention to Response Task, SART [38]) instead of higher-level cognitive measures for two main reasons. First, attention is at the core of the mechanisms in theories describing SICI [2,3,7,10]. Second, SART is capable of detecting brief lapses of attention due to the short response windows, and that way, it can be sensitive to even subtle influences on cognitive performance.

## 2. Material and methods

The stage 1 protocol for this Registered Report was approved on 26 June 2024. The protocol, as accepted via PCI Registered report, can be accessed at https://osf.io/5sdbp. Deviations from the accepted protocol are shared in electronic supplementary material, table S1, which is accessible on the OSF page of the project at https://osf.io/zr3y5. A track changes version of the manuscript is available at https://osf.io/krqmz. Data, analysis scripts and other materials are available at https://osf.io/7ufkw/. The research plan was approved by the Institutional Review Board at Eötvös Loránd University (registration number 2024/299) and complies with all relevant ethical regulations.

### 2.1. Design and procedure

The experiment was conducted online (for details on sampling see the *Subject Recruitment* section). After accepting the informed consent form, participants received instructions to avoid any non-experiment-related activities, such as listening to music or chatting. Throughout the experiment, participants engaged in three main types of activities: the answering of survey questions, experimental manipulation and sustained attentional performance measurement.

Participants were randomly assigned to one of two conditions: a scarcity salience condition or a control condition. We utilized words and expressions as cues in the manipulation. In both conditions, participants were shown three cues on their screen. In the financial salience condition, the cues were financial scarcity-related, while in the control condition, they were financially neutral cues. To investigate whether cues related to relative scarcity or cues related to absolute scarcity deteriorate sustained attentional performance, we assembled two types of financial scarcity-related cues: absolute-scarcity-related (*absolute* cue) and relative-scarcity-related (*relative* cue) words and expressions. Each batch of three cues presented in the experimental condition contained only either relative or absolute cues. With all words visible on their screens at the same time, participants were instructed to generate one association for each of the presented cues. Each participant repeated this part of the procedure twice. Participants in the experimental condition either encounter both absolute and relative cues in the two rounds (with their order counterbalanced) or the same type of cues in both rounds (absolute-absolute, relative-relative), resulting in three subconditions with an even chance of occurrence. There was no time constraint on this task. After the manipulation was concluded, participants completed the *Sustained Attention to Response Task* (SART [38]). Immediately after the SART is concluded, participants were asked to indicate their current level of financial worrying and whether they experienced any scarcity- or abundance-related thoughts during the attention task.

**Figure 1.**
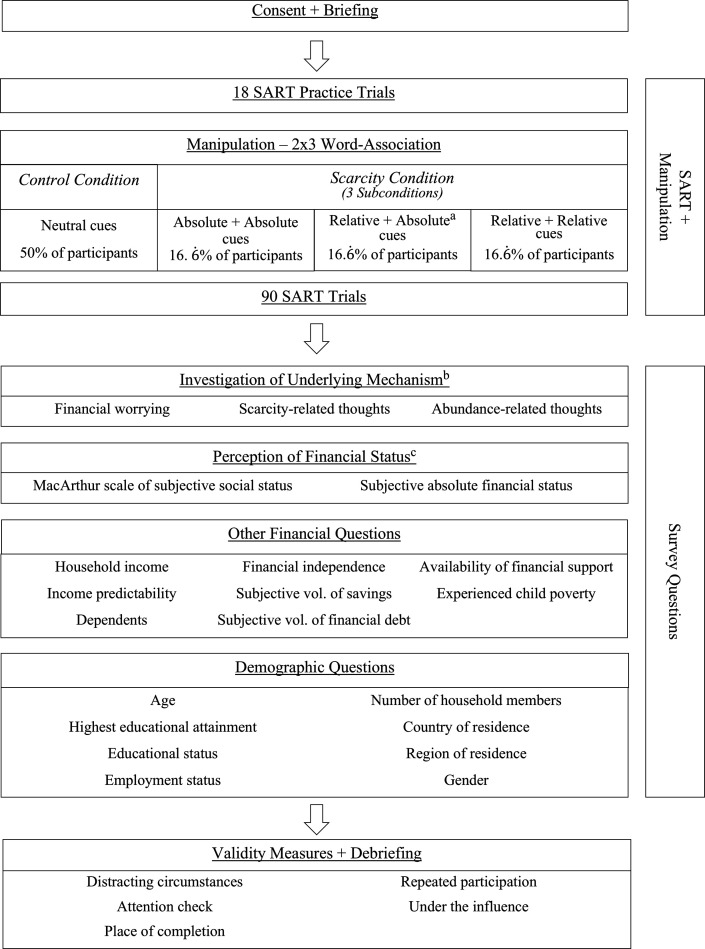
Experiment flow diagram. The used measures are discussed in the ‘Measures’ section in detail. ^a^The order of the relative and absolute groups of cues was randomised. ^b^The order of the financial worrying, scarcity-related thoughts and abundance-related thoughts were randomized, ensuring that the latter two always followed each other. ^c^The MacArthur scale of subjective social status and the subjective absolute financial status were asked in a randomised order.

Next, participants were asked to respond to items about their socioeconomic context and demographic background (see the full list in the *Measures* section). They were required to respond to all questions. To avoid carryover effects, the items investigating the perception of financial status were asked first in a randomized order. Finally, participants were asked to answer a number of questions regarding the circumstances of their participation, after which they were debriefed. The procedure is summarized in figure 1.

### 2.2. Experimental materials

#### 2.2.1. Stimuli in the scarcity salience condition

The stimuli have been selected through the use of mixed methods. Our goal was to create words associated with absolute (related to the lack of money, poverty) and relative financial scarcity (related to things that are not necessary for getting by but might be desired and unattainable due to financial constraints, abundance). First, to achieve a wide pool of potential stimuli, we collected words and expressions using a Large Language Model (LLM).^4^ We created 32 different prompts mixed in Hungarian and English. All prompts were structured as follows: *Write 100 words that make people think of* […]. *Write down words that occur in everyday conversation. Write all the words on one line and separate them with commas. Don't write anything else down, just the words and the commas!* Only the expressions in the brackets changed across prompts, e.g. poverty, wealth and expenditure. The model was allowed to utilise up to 1000 tokens (approx. 4000 characters). The complete list of prompts is made available in electronic supplementary material, table S2 of the supplementary materials. The list of expressions provided by the LLM can be found on the OSF page of the project (at https://osf.io/zvy39).

As a next step, one of the authors selected the relevant responses by reducing redundancy and removing seemingly non-scarcity-related expressions and words. This process resulted in a list of 207 words and expressions (available at https://osf.io/uqs4b). Subsequently, two authors used this list to collaboratively compile 15 expressions both for relative and absolute scarcity subconditions. The final lists of selected expressions can be found in table 2.

**Table 2.** Experimental stimuli used in the experiment.

financial cues	control cues
*absolute cues*	
insolvency	way of thinking
social aid reduction	Acacia trees
utility charge	fib
financial constraints	deep dream
food expenses	basketball championship
rent	linen
unexpected expenses	dewy mornings
debt repayments	compulsion to conform
too low income	deep root system
can't afford	not resourceful
inflation	air
unpaid bills	complicated stories
fuel costs	cold water tap
clothing expenses	long explanation
financial insecurity	with full chest width
*relative cues*	
passive income	falling feather
American holiday	whimsical weather
city centre property	hot concrete
brand new mercedes	terrible singing
authentic gucci	the exact opposite
comfortable lifestyle	dizzying height
housekeeping service	land mammal
villa	exclamation mark
taxi service	making friends
wealthy	respectful
heated pool	frequent watering
original Rolex	a close bond
financial freedom	pale painting
financial security	attentive audience
investments	bushies

Note. The English cues were not used in the experiment. They were translated to support the reproducibility of our research. Electronic supplementary material, table S3 contains the original, Hungarian cues along with their English translations.

#### 2.2.2. Stimuli in the neutral condition

The cues in the neutral condition were generated as follows. First, two assistants were instructed to independently generate expressions that are unrelated to relative and absolute scarcity and are not easier or harder to understand than the words and expressions selected for the scarcity salience condition.^5^ As a result, 41 expressions were collected (see the OSF page of the project at https://osf.io/v76y8). Next, using this list as a starting point, one of the authors created a list of 30 cues. In the final list, each word is paired with a scarcity-related cue to match its grammatical structure with regard to the number of words and word class. The final lists of generated expressions can be found in table 2.

#### 2.2.3. Validation of the selected stimuli

In addition to face validation, we aimed to evaluate the semantical distance of scarcity-related and neutral cues from our target concepts. To do that, we calculated the cosine similarities between (i) the absolute-scarcity-related cues as well as their neutral counterparts from the expression ‘financial scarcity’ and (ii) the relative-scarcity-related cues as well as their neutral counterparts from the expression ‘financial abundance’ using the word embedding ‘text-embedding-ada-002’ [41]. Cosine similarity indicates the level of semantic similarity between words by measuring the cosine of the angle between the vectors of the semantic meanings of words in a word embedding (see footnote 3). Higher numbers indicate higher semantic similarity between words. The similarities were calculated using the translated English cues, as the embedding we applied has greater accuracy in English. In line with our expectations, the cosine similarities of scarcity cues indicate higher similarity with the target concepts (*M* = 0.793, s.d. = 0.049) than the neutral cues (*M* = 0.748, s.d. = 0.019). Such 0.450 average difference is notable: for comparison, using the same word embedding, the similarity between ‘dog’ and ‘cat’ is 0.864, while between ‘dog’ and ‘statistics’ is 0.800. Electronic supplementary material, table S4 includes the cosine similarity scores of the cues.

#### 2.2.4. Assessment of sustained attention

We opted to investigate SICI by focusing on sustained attention for two main reasons. First, attention is at the core of the mechanisms in theories describing SICI [2,3,7,10]. Second, we expect that, contrary to higher-level cognitive performance measures (e.g. IQ tests), by measuring sustained attention, we could more sensitively assess the subtle influence of scarcity-related cues and even detect brief lapses of attention.

To measure sustained attention, we asked participants to complete the Sustained Attention to Response Task (SART [38]). SART is a frequently used and validated tool to investigate attentional performance, attentional lapses and mind wandering (e.g. [42]). For our study, we adapted the original design of Robertson *et al.* [38] in jsPsych [43]. The SART has one property that makes it the ideal choice of measurement tool for sustained attention for our design. Unlike similar tasks where sustained attention is fundamental, such as the Flanker, Stroop or categorisation tasks, which were primarily used in the literature to measure cognitive performance [2,19,23], here participants only need to use one button, simplifying task instructions and making it easy to complete it using smart devices, rendering it ideal for online data collection.

**Figure 2.**
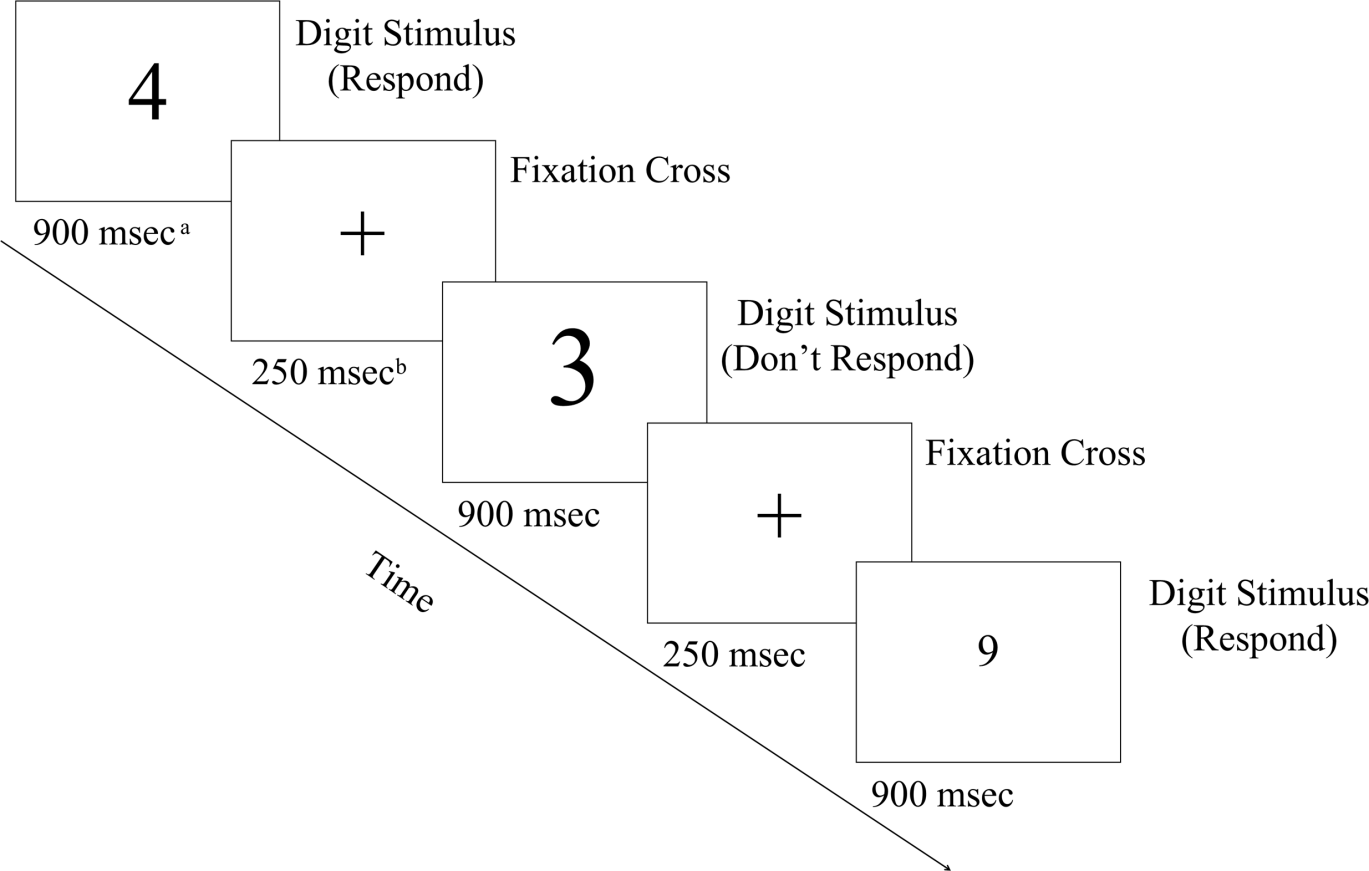
Flow diagram of the sustained attention to response task. ^a^900 ms is the maximum display time; if a participant responds sooner, the digit disappears immediately. ^b^250 ms is the minimum display time, plus any time saved from an early response to the previous digit.

During the task, participants were presented with one-digit integers [1–9]. As they had the flexibility to participate using any computer or smart device, the font size of the integer changed randomly between 6, 8, 10, 16 and 20 viewport heights which represent the size of the digits relative to the vertical size of the screen in percentage units. The original design used exact (and not relative) font sizes, but here we collected data on devices with varying screen sizes so the constant font sizes would not cause inconsistencies during the experiment. At the bottom of the screen, a button with the label ‘Continue’ was present during the integer trials. The participants’ task was to press the button each time a new integer appeared (known as ‘go trials’), except when the number ‘3’ was displayed (known as ‘no-go trials’). Participants encountered 90 trials in total, in a way that each integer was presented 10 times in a random order. We pseudo-randomised the order of the digits in a way that trials displaying number ‘3’ could not be neighboured by another such trial. The integers were programmed to be visible for 900 ms and disappeared once a response was provided or the time ran out. Participants could react at any time during the 900 ms the integers were presented. A fixation cross was displayed between the presentation of integers. It was programmed to be shown for a minimum of 250 mss, along with any remaining time from the 900 ms allocated for responding to the previous digit stimulus. For example, if a participant responded to the digit in 800 mss, the fixation cross was programmed to be visible for 350 mss. The task flow is depicted in figure 2.

### 2.3. Measures

During the course of the experiment, we collected self-report data related to three different topics: finances, demographics and response validity (see the OSF page of the project for the Hungarian and English-translated versions of the Qualtrics survey at https://osf.io/qjzgd/). For clarity, we also provided the descriptions of the variable transformations that will be made during the analysis.

#### 2.3.1. Sustained attention measures

*Accuracy*. We categorized participants’ responses in the go and no-go trials in different ways. A trial was coded as missed (‘0’) if the button was pressed in no-go trials or was not pressed in go trials. The trial was coded as correct (‘1’) if the button was not pressed in go trials or pressed in no-go trials. For the analysis, we aggregated this variable on the participant level for go and no-go trials separately, resulting in two count variables ranging from 0 to 80 (go trials) and 0 to 10 (no-go trials). We utilized trial-level data for the outlier exclusion in the robustness analysis.

*Response Time*. In go trials, we measured the time passed between the appearance of digit stimuli and the button press made in response in milliseconds.

#### 2.3.2. Financial measures

*Availability of financial support*. We solicited data from participants on their availability of social support by asking them to indicate whether they have any family, friends or other individuals they could turn to for help in case of financial struggles (‘Yes’, ‘No’).

*Dependents*. Participants indicated whether anyone is dependent on their income (‘Yes’, ‘No’, ‘I don’t have income’). We created a dummy variable to distinguish between individuals with dependents and those without dependents or no income.

*Experienced child poverty*. Following Ng *et al.* [44], participants were asked to indicate their perceived relative financial status of their family during their childhood on a seven-point categorical scale (‘A lot worse off than others’, ‘Worse than others’, ‘A little worse than others’, ‘Average’, ‘A little better than others’, ‘Better than others’ and ‘A lot better off than others’). We created two dummy variables to represent perceptions of childhood financial status: one for worse than average and one for better than average. Additionally, we Helmert coded^6^ the variable.

*Financial independence*. Participants were asked to indicate their level of financial dependence on others (‘Yes, I am completely dependent on others financially’, ‘Yes, I partly depend on others financially, but I could get by on my own’, ‘I am completely independent financially’). We Helmert coded this variable in the analysis.

*Household income*. Participants rated their household’s net monthly income on a scale ranging from less than 50,000 HUF (approx. 140 USD) to more than 1 500 000 HUF (approx. 4100 USD), divided into 30 equal increments and the final bracket for incomes exceeding 1 500 000 HUF. Following the official measure of OECD for household income [45], we transformed the income measure by dividing it by the square root of the number of household members, in order to adjust for the number of individuals utilizing the income.

*Income predictability*. We asked participants to indicate their perceived predictability of income on a 101-point scale (0–100), with endpoints of ‘Not at all predictable’ to ‘Completely predictable’.

*MacArthur scale of subjective social status*. MacArthur scale of subjective social status [46] measures an individual’s perceived social status in relation to others in their society. We used a modified version, in which we specifically inquire about financial status. This version of the scale consists of a ladder graphic with 10 rungs, with the top rung representing the richest and the bottom rung representing the poorest. Participants were asked to indicate the rung that they perceive themselves to be on, in comparison to others in society.

*Subjective absolute financial status*. We asked participants to indicate their level of agreement with the statement ‘I have enough to get by’ on a 101-point scale, ranging from ‘I do not agree at all’ to ‘I agree completely’. For different measures, see Ravallion [47] or Kuivalainen [48].

*Subjective volume of financial debt*. Following Lusardi & Mitchell [49], we asked participants to indicate their perceptions regarding the degree of their indebtedness on a 5-point Likert scale ranging from 1 (‘Completely disagree’) to 5 (‘Completely agree’). We Helmert coded this variable in the analysis.

*Subjective volume of savings*. We asked participants to indicate the amount of their savings on a three-point scale (‘No, I have no savings.’, ‘Yes, I have little savings’, ‘Yes, I have a lot of financial savings’). We Helmert coded this variable in the analysis.

#### 2.3.3. Underlying mechanisms

*Scarcity-related thoughts*. Participants were asked to indicate whether, while completing the SART, they experienced any thoughts about their financial situations that they feared they would encounter (‘Yes’, ‘No’).

*Abundance-related thoughts*. Participants were asked to indicate whether, while completing the SART, they experienced any thoughts about their financial situations that they wished they would encounter (‘Yes’, ‘No’).

*Financial worrying*. We measured participants’ momentary financial worrying with a modified version of the first item on the Financial Anxiety Scale [50]. Participants were asked to indicate on a Likert scale ranging from 1 (‘Completely disagree’) to 7 (‘Completely agree’) how much they agree with the statement, ‘I currently feel anxious about my financial situation’.

#### 2.3.4. Demographic measures

*Age*. Participants were requested to select their birth year from a dropdown list.

*Country of residence*. Participants were asked whether they predominantly reside in Hungary (‘Yes’, ‘No’).

*Educational status*. Participants were asked about their current enrolment status in full-time education (‘Yes’, ‘No’).

*Employment status*. Participants were requested to specify their current primary employment status (‘Full-time (not entrepreneur)’, ‘Part-time’, ‘Entrepreneur’, ‘Unemployed’, ‘Retired’, ‘Other’). We created dummy variables using ‘Full-time (not entrepreneur)’ and ‘Other’ as reference groups.

*Gender*. Participants were asked to indicate their gender. The available options align with the Hungarian language, which uses a single term that encompasses both sex and gender identity (‘Female/Woman’, ‘Male/Man’, ‘Nonbinary’). We created dummy variables using ‘Male/Man’ as the reference group.

*Highest educational attainment*. Participants were asked to indicate their highest completed educational level (‘Less than elementary school’, ‘Elementary school’, ‘Skilled worker qualification’, ‘High school’, ‘Vocational education’, ‘BA/BSc’, ‘MA/MSc’, ‘Doctorate or higher’). We created a dummy variable to distinguish between those whose highest completed education was high school and those who completed more. This dummy was used in the main analyses only. In the exploratory analyses, we used a more fine-grained Helmert-coded version of this variable.

*Number of household members*. Participants were requested to indicate the total number of individuals in their household, including themselves. To prevent typos, we did not permit numbers greater than 15.

*Region of residence*. Participants were asked to indicate their region of residence (‘Rural’, ‘Urban’, ‘Other’). We created dummy variables using ‘Rural’ as the reference group.

#### 2.3.5. Validity measures

*Attention check*. To identify inattentive survey respondents, we asked participants the following question: ‘This question is designed to test participants’ attentiveness. To demonstrate that you are paying attention, please do not answer this question. If you have accidentally selected an option, you can unclick your answer’. This item is a modified version of the item used by Paas & Morren [51].

*Device type*. We recorded participant browser metadata to determine their device type (a computer or a phone/tablet) using jsPsych. We created this indicator to account for the difference in response means between computer users (mouse/touchpad input) and smart device users (screen touch input).

*Distracting circumstances*. To minimize the noise in our data, we asked participants the following: ‘Were there any distractions in your environment that drew your attention during participation or made your task harder in any other ways, e.g. condition of your device, loud noises?’ (‘Yes’, ‘No’).

*Place of completion*. Participants were asked to disclose their location during the completion of the study (‘At work’, ‘At home’, ‘On the street/on a means of transport/in a waiting room’, ‘Other’). We created two dummy variables to distinguish participants who complete the experiment during travel or work from those who complete it at home.

*Received compensation*. We recorded if the participant received any compensation (‘Yes’, ‘No’). For details regarding the different compensation structures, see the *Subject Recruitment* section.

*Repeated participation*. To minimize the probability of having multiple observations from the same people, we asked participants to indicate whether they had participated earlier in the experiment (‘Yes’, ‘No’).

*Under the influence*. Participants were asked to indicate if they were under the influence of drugs or alcohol at the time of participation (‘Yes’, ‘No’).

### 2.4. Data sampling

#### 2.4.1. Stopping rules and power analysis

Based on the availability of our resources, our aim was to collect 3000 participants after the exclusion criteria were applied. This target sample size is larger than the sum of participants from all prior studies we could identify. Using the R [52] package *pwr* [53], we determined that sample size would be sufficient to provide evidence for an effect size of 0.14 Cohen’s *d* in a model with 7 predictors [54], at the 0.05 significance level with 0.80 power.

Additionally, to examine how the impact of financial scarcity-related cues on sustained attentional performance varies across different demographic dimensions, we aimed to recruit a diverse range of Hungarian subpopulations, encompassing various socioeconomic groups. Beyond reaching our target sample size, we committed to recruit at least 50 participants in 24 subgroups varying in their level of income and education to increase the generalizability of our results (i.e. quota sampling; see [55]). We determined the minimum per group sample size based on the anticipated accessibility of the hardest-to-reach subgroups (e.g. people living above very good living standards who did not finish high school). The descriptions of the defined groups are shared in the *Recruitment Group Descriptions* section of the electronic supplementary material.

#### 2.4.2. Subject recruitment

To recruit participants, we adopted a three-step approach. First, via collaboration with editors and journalists, we advertised our call for participation at regional and national news sites. In total, two news sites published our call, and the links to these articles are shared in the electronic supplementary material, table S5. By leveraging these platforms, we expected to reach a wide audience with diverse socioeconomic backgrounds (for a similar approach, see our prior work [56]). Second, we conducted data collection with a snowball method, by posting our call for participants on different social media sites.^7^ The advertisements and their performance metrics are shared in the electronic supplementary material, figures S1, S2 and table S6. Participation through these two channels was on a voluntary basis, with no compensation. Finally, as we could not reach our target sample size of at least 50 participants in all identified groups, we collaborated with a data collection agency to complement these samples. This enabled us to near the sample size we aim to collect and ensure adequate representation of participants from diverse layers of the population. Participants recruited via our collaboration with the data collection agency received HUF 300 (approx. USD 0.78) in exchange for their participation.

### 2.5. Registered data analysis strategy

The main conclusions of the paper are based on the outcome of the *main analyses*. In these analyses, we examine whether the deteriorating effect of financial scarcity-related cues on sustained attentional performance increases as a person’s poverty increases (SICI). Next, in the *exploratory analyses*, we explore the generalizability of SICI and identify which characteristics of the individuals and scarcity-related cues moderate its strengths. The *robustness* of both the main and exploratory analyses was assessed using a multiverse analysis approach.

### 2.6. Statistical framework for inferences

Throughout the analysis, we used 0.05 as the alpha level within the frequentist framework. While testing the main hypothesis,^8^ in case of non-significant results, we used equivalence testing [57] to determine whether we can present evidence against the alternative hypothesis. We computed a 90% confidence interval for the predicted effect size and assessed whether (a) the interval falls below the effect size we can realistically detect based on the power analysis or (b) it encompasses it. In turn, we drew the following conclusions: (a) data supports the absence of the effect or (b) data is inconclusive regarding the absence or the existence of the effect. In case the *p*-value was significant, and the direction of the interaction aligned with our hypothesis, we treated it as evidence for the alternative hypothesis. If the *p*-value was significant but the direction of the interaction was misaligned with our predictions, we treated it as evidence against the existence of SICI.

### 2.7. Data preparation for the analyses

#### 2.7.1. Attentional performance index

For each individual, we created an *attentional performance index* based on their SART results. To do that, we divided the accuracy in the no-go trials by the mean response time in the go trials, as has been done in prior work (e.g. [58,59]). Consequently, we aggregated data at the participant level. Calculating the index enabled us to measure attentional performance while also controlling for individual differences in speed-accuracy trade-off preferences [42]. These preferences need to be accounted for, as participants in the SART are asked to be as fast and as accurate as possible. Since it is not possible to do both at the same time, participants need to optimize between these two strategies [60]. That way, measuring only accuracy or response time alone could not fully capture attentional performance. To punish missed trials, we added 900 ms per missed trial, which is the maximum response time one can have in a trial. Such attentional performance index was shown to be sensitive to differences in the frequencies of momentary lapses of attention [42]. We also reported response times in the go trials and the number of errors in the no-go trials and used them as outcome measures in the robustness analysis.

#### 2.7.2. Poverty index

While financial scarcity is conceptualized in the literature as a multidimensional measure (e.g. subjective versus objective and relative versus absolute [26], in most empirical investigations of SICI, absolute objective scarcity was measured and tested whether it moderates the effect of scarcity-related cues on cognitive performance [9]. To match the applied methods with the theoretical predictions, we aimed to utilize absolute objective poverty, absolute subjective poverty and relative subjective poverty in testing our hypothesis. Given the limited evidence in the literature, we believed this would enable us to have a more sensitive test of our hypotheses.

We created a poverty index by standardizing and averaging with equal weights three measures capturing different dimensions of poverty: the adjusted household income (absolute objective poverty), the score of the MacArthur scale (subjective relative poverty) and the self-reported financial status (subjective absolute poverty), multiplied by −1. As the different measures of poverty often do not correlate heavily [61], we also fitted models using the individual measures. For details, see the *Assessing the Robustness of the Main Analysis* section.

### 2.8. Main analysis: SICI in the general population

To reveal whether the deteriorating effect of scarcity-related cues is moderated by individuals’ poverty levels, we fitted a linear regression model with the attentional performance index as the outcome variable and included the experimental condition dummy, the poverty index, the interaction term between the condition dummy and the poverty index, the highest education attainment dummy variable distinguishing those who have a higher education completed is higher than high school and those who do not,^9^ age, whether the participant received monetary compensation, and the device used by participants as predictors. To ensure data quality and validity, we applied the following exclusion criteria.

— Participants who did not complete the questionnaire were excluded from the analysis.
— Participants under the age of 18 were excluded from the study.
— Participants who self-reportedly participated in the study previously were excluded to avoid duplication of data.
— Participants who were self-reportedly under the influence of any drugs or alcohol during test completion were excluded.
— Participants who answered the attention check incorrectly were excluded.
— Participants providing nonsensical responses in the association task were excluded to maintain data quality. To evaluate the validity of individual data, we coded manually whether none of the six answers submitted to the association task during the manipulation was a meaningful word. In addition to those where all words were incomprehensible, we also excluded responses containing three consecutive repetitions, as well as participants who predominantly used numbers or punctuation marks.^10^
— Participants with an accuracy rate below 60% in the go trials or those who made more than 8 errors in the no-go trials were excluded. We introduced these criteria to exclude participants who may have answered randomly or inattentively.
— Participants who took more than 10 min or more between the start of the association task and the completion of the first experimental trial were excluded.

Additionally, to account for outliers, we conducted a 99% winsorization on the attentional performance index and response time outcome variables.

#### 2.8.1. Assessing the robustness of the main analysis

To assess the analytical robustness of our findings, we conducted multiverse analyses. A multiverse analysis is an analytical method that ‘involves performing all analyses across the whole set of alternatively processed data sets corresponding to a large set of reasonable scenarios’ [62]. We have chosen this method because we acknowledge the presence of multiple decision points within the analytic process (as detailed below), where various choices are justifiable and have the potential to impact the results significantly. Accordingly, we systematically conducted all possible combinations of the main analysis with the following alternative specifications: exclusion criteria (2 variations), outlier treatment (2 variations), the outcome variable (3 variations), the used poverty indicator (4 variations) and the inclusion of secondary control variables (2 variations). Note that there are two addenda when the outcome variable is the number of errors in the no-go trials: (i) we did not conduct one of the outlier treatments (winsorisation) as it would not make sense, given that the number of errors is discrete (ii) to comply with our pre-defined analysis plan, we used the number of errors as the outcome variable in our analysis after verifying that fewer than 60% of participants made one or zero errors. As a result, the multiverse approach yielded a total of 80 analyses (including the main analysis). In the following, we discuss the alternative specifications.

*Exclusion criteria*. We repeated all assessments using two distinct sets of criteria. The first set was the same as detailed in the main analysis section. In the second set, we implemented a more stringent set of criteria, which will differ from the first one in the following ways: participants scoring less than 80% accuracy in go trials, those making more than 6 mistakes in no-go trials and those committing more than 10 consecutive errors were excluded, as well as those who indicated that they are not spending the majority of their time in Hungary.

*Outlier treatment*. We repeated all analyses with winsorization, as described in the main analysis section, as well as with no outlier exclusion. As mentioned above, winsorization was not applied when the outcome variable was the number of errors in the no-go trials.

*Outcome variable*. While the main analysis relied on the attentional performance index, a composite of response time and accuracy, the multiverse analysis incorporated the number of errors in the no-go trials and the mean response time in the go trials as alternative outcome variables. In case of the number of errors, Poisson regression models were fitted. As mentioned above, we included only the number of errors as an outcome measure if a ceiling effect was not detected.

*Utilised poverty indicator*. We fitted models using each of the financial indicators (adjusted household income, MacArthur scale score, subjective absolute poverty) that make up the poverty index.

*Control variables*. We conducted analyses both with and without the inclusion of the following variables: age, whether the participant received monetary compensation, and device dummy. We opted to keep the highest educational attainment dummy in all models because, due to its presumed high correlation with socioeconomic status, it may act as a confounding factor, and removing it is expected to amplify the detected effect of socioeconomic indicators in our analyses.

### 2.9. Exploratory analysis: generalizability of SICI

In the exploratory analysis, to identify individual and cue characteristics that are moderating SICI, we fitted LASSO (Least Absolute Shrinkage and Selection Operator) regression models within a stability selection framework [63]. LASSO regression is a feature selection method that proves particularly beneficial when dealing with a relatively high number of potential predictor variables. LASSO regression utilizes regularization to eliminate irrelevant variables from the model by shrinking their coefficients toward zero. The stability selection method involves iteratively fitting the LASSO model on a subset ($\frac{n}{2}$) of the data a number of predetermined times, and always selecting the same predefined number of predictors. After completing all iterations, the relative importance of variables is determined by the number of times they were selected. This approach was shown to decrease false selection rates and improve accuracy compared to the use of a single LASSO regression in case of high multicollinearity in the data [63,64]. We utilized the complementary pairs stability selection with improved error bounds as the selection method because, using this method, the choice for the number of selected predictors in the individual LASSO regressions has negligible impact on which variables are selected [65].

To execute the analysis, we utilized the R package *stabs* [66] to conduct 50 iterations of LASSO regression model fitting and select the top 10 predictors during each run. Note that the penalty parameter of the LASSO regression models was chosen automatically so that 10 variables were selected. We considered a variable stable (i.e. a robustly important predictor) when its probability of being selected by the algorithm is at least 0.6. This probability is customarily set between 0.6 and 0.9 [64]. We opted to use the lower bound of this interval as we expect high multicollinearity and small effects, and therefore, relatively high variance in the selected variables.

In the LASSO models, unlike in the main analysis, we included adjusted household income, MacArthur scale score, subjective absolute poverty score, debt, savings, availability of financial support, income unpredictability, childhood financial status, financial dependents and being financially dependent as predictors. Additionally, we included dummy variables indicating the presence of absolute and relative scarcity-related cues during manipulation, as well as their interaction. Finally, we included all collected demographic variables. All plausible predictors, including two- and three-way interactions, were added to the model. We applied the same exclusion criteria as in the main analysis of attentional performance. The variables we included in the LASSO regression model are summarized in table 3.

**Table 3.** Main effects and two-way interactions in the LASSO models.

main effect	two-way interaction
condition	condition : poverty index
poverty index	condition : adjusted household income
adjusted household income	condition : subjective absolute poverty
subjective absolute poverty	condition : MacArthur score
MacArthur score	condition : savings
savings	condition : financial debt
financial debt	condition : experienced child poverty
experienced child poverty	condition : income predictability
income predictability	condition : dependents
dependents	condition : financial independence
financial independence	condition : availability of financial support
availability of financial support	condition : employment status
presence of absolute cue	presence of absolute cue : relative cue
presence of relative cue	presence of absolute cue : adj. household income
gender	presence of relative cue : MacArthur score
age	
received compensation	
highest educational attainment	
employment status	
educational status	
device type	
distracting circumstances	
place of completion	

Note. Certain categorical variables were Helmert- or dummy-coded before the analysis: see the Measures section for details. Due to the high number of three-way interactions, they are not shared in this table. The Three-Way Interactions are shared in the *Three-Way Interactions in the Exploratory Analysis* section of the electronic supplementary materials.

#### 2.9.1. Assessing the robustness of the exploratory analysis

We used a multiverse approach to test the analytical robustness of the exploratory analysis with the same combination of choices as described in the robustness test of the main analysis with the exception of two alternative specifications: the inclusion of control variables and the utilized poverty indicator. These two alternative specifications are meaningless in the context of the LASSO method. This systematic exploration yielded a total of 10 analyses (including the main exploratory analysis).

### 2.10. Survey attrition

Since most participants got involved in our research on a voluntary basis and without any financial incentive, we anticipated high survey attrition and careless responding rates. Our main analysis based on SART performance might lose its sensitivity to detect SICI if financially disadvantaged individuals in the scarcity salience condition drop out more often or pay less attention to the instructions than their richer counterparts. It should be noted, however, that this pattern of unequal rates of attrition or instruction adherence might also be evidence for SICI, as it would demonstrate that poorer people find it harder to stay on task in the presence of scarcity-related cues.

One-sided group comparisons between the experimental and control conditions were used to test whether participants with worse financial backgrounds were more likely to quit the experiment early or get excluded due to inattentiveness or poor performance in the scarcity condition. After making sure that assumptions of normality and variance homogeneity held by checking if kurtosis and skewness were not outside of –10 to 10 and –3 to 3, respectively, and running a Brown–Forsythe test to assess variance homogeneity, poverty index, adjusted household income and subjective financial status were compared using independent samples *t*-tests. MacArthur scale scores were compared using the Mann–Whitney–Wilcoxon test. Bonferroni correction was applied to control for the family-wise error rate. We repeated this analysis with both sets of exclusion criteria.

### 2.11. Investigation of underlying mechanisms

#### 2.11.1. Current financial worry

To test whether participants assigned to the scarcity condition experienced elevated levels of financial worrying, we fitted ordinal logistic regression models with the same parameters that we will use in the primary analysis. Additionally, we repeated the same robustness analysis as described for the primary analysis, with two differences: we did not use different outcome measures, and we did not apply winsorization on the outcome measure.

#### 2.11.2. Scarcity-related thoughts

To test whether participants assigned to the scarcity condition had scarcity-related thoughts more frequently, we fitted three binomial logistic regression models with the same parameters that we will use in the primary analysis. With the first model, we investigated if the effect of the experimental manipulation on the likelihood of thinking about any type of scarcity-related cues is moderated by the poverty index. With the second model, we tested whether absolute cues were more likely to evoke thoughts about scarce financial situations than relative or control cues. With the third model, we investigated whether relative cues were more likely to evoke thoughts about desired financial situations than absolute or control cues. Additionally, we repeated the same robustness analysis as described for the primary analysis, with two differences: we did not use winsorisation nor different outcome measures.

## 3. Results

### 3.1. Data

A total of 7947 participants began the experiment, with 5658 recruited through news outlets, 1613 via Facebook ads and 676 through the panel provider. In addition to the predefined exclusion criteria, we removed additional observations. Thirty-seven participants opened the survey following the cognitive tests more than once. In the case of these participants, we only took into consideration the try that was finished first. Another 23 participants’ data was corrupted during data collection as no SART data was recorded during the session due to an unknown software issue. These participants most probably solved the SART task without complications, as the preceding and subsequent questionnaires were collected successfully. However, the SART data was empty, and it was not possible to retrieve them from the JATOS server. We excluded these participants from data analysis. After applying the predefined exclusions, 4280 participants remained (3885 from news outlets and Facebook and 395 from the panel). Using the more stringent set of exclusion criteria, 3870 participants remained in the final sample. Descriptive statistics of the primary sample are shared in table 4. The number of participants meeting each exclusion criterion is shared in the electronic supplementary material, tables S7 and S8 for the primary and more stringent sets of exclusion criteria, respectively.

**Table 4.** Overall and Group-Specific Descriptives.

variable	overall (*n* = 4280)	scarcity (*n* = 2147)	control (*n* = 2133)	test
*primary scarcity measures*				
poverty index	0 (0.839)	−0.017 (0.835)	0.017 (0.843)	1.327 (0.185)
adjusted household income (HUF 1000)	541.351 (307.971)	546.641 (308.299)	536.026 (307.621)	−1.128 (0.26)
subjective relative poverty	5.628 (1.76)	5.659 (1.756)	5.596 (1.764)	−1.157 (0.247)
subjective absolute poverty	71.52 (27.343)	71.952 (27.118)	71.084 (27.566)	−1.039 (0.299)
*demographic characteristics*				
has dependents (%)	58.201	58.78	57.618	0.546 (0.46)
highest education: high school (%)	14.322	13.414	15.237	2.75 (0.097)
age	46.096 (12.081)	46.579 (12.011)	45.609 (12.134)	−2.631 (0.009)
*participation characteristics*				
distracted (%)	21.332	21.192	21.472	0.035 (0.852)
compensated (%)	9.229	9.874	8.579	1.989 (0.158)
participated while travelling (%)	3.014	2.748	3.282	0.868 (0.351)

Note: This table presents means and percentages for each variable discussed in the main text. Standard errors are in parentheses. Test column reports the test statistics and the *p*-values of two-sided *t*-tests for continuous variables and chi-squared tests for binary variables. Data include only participants who met inclusion criteria. Income is estimated using the lower bound of the reported income range. Only age differs significantly in the two groups, though the effect size is negligible (*d* = 0.08). A more detailed list of descriptive statistics is available in electronic supplementary material, table S9.

Though we reached our target sample size of 50 participants for the majority of predefined demographic cells having at least a high school degree, we ran out of resources before being able to reach the target everywhere. Table 5 presents the sample sizes after exclusions for each demographic group.

**Table 5.** Sample sizes by income level and highest educational attainment.

income range (1000 HUF)	highest educational attainment				*total*
	less than high school	high school	vocational education	university or higher	
–109.7	30	82	44	220	*376*
109.7–139.9	11	37	26	132	*206*
139.9–191.7	19	74	46	252	*391*
191.7–258.4	18	98	61	494	*671*
258.4–371.1	12	73	53	649	*787*
371.1–	22	137	94	1596	1849
*total*	*112*	*501*	*324*	3388	

Note. Income is estimated by dividing the lower bound of the reported income range by the number of household members.

### 3.2. Main analysis: SICI in the general population

**Figure 3.**
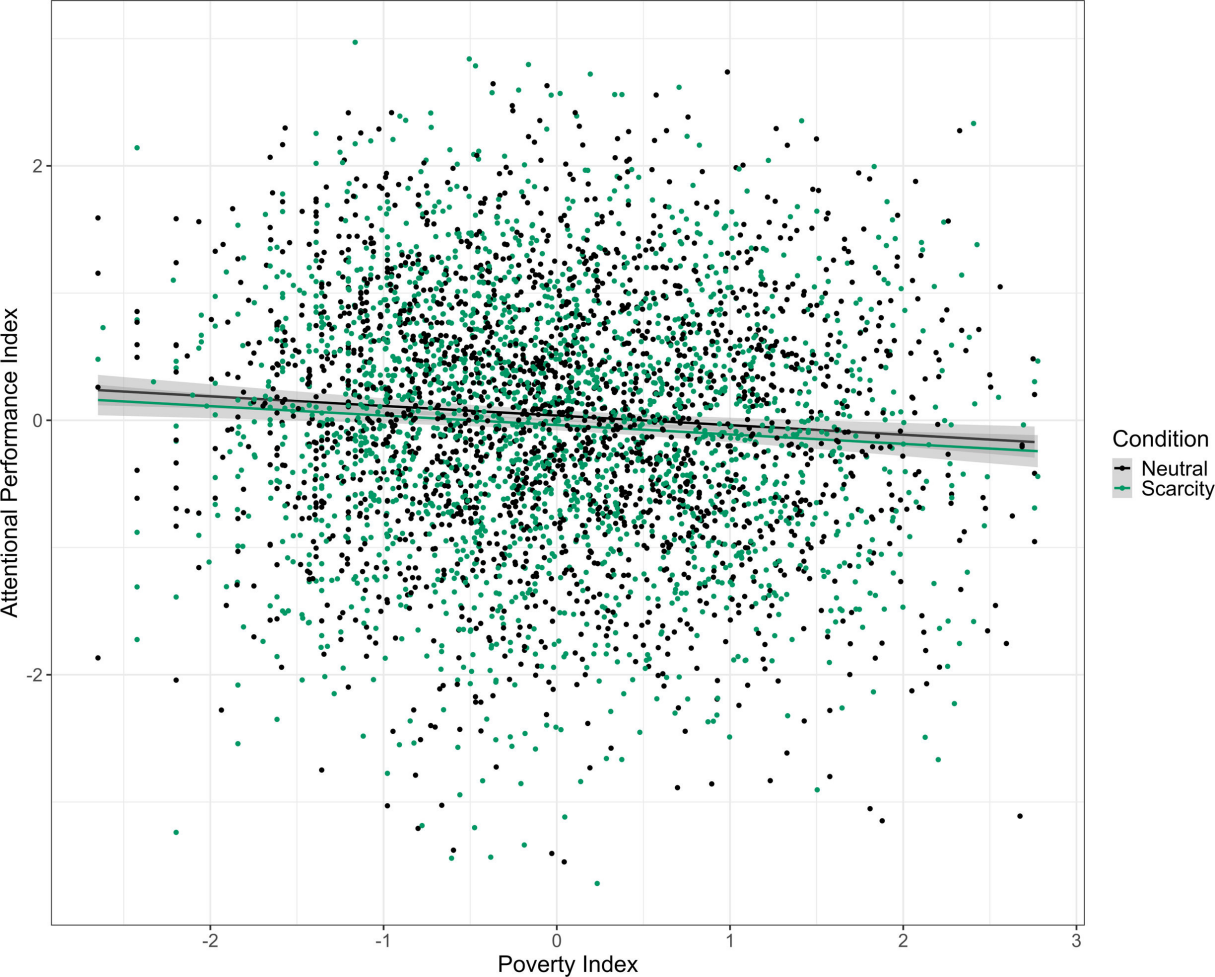
Scatter plot of poverty index and attentional performance index by experimental condition. Both measures are mean-centred and standardised. The shaded grey areas around the regression lines represent 95% confidence intervals.

The regression analysis provided evidence against the effect being larger than 0.14 *d* (*β* = 0.007 [−0.045, 0.060], *d*_transformed_ = 0.015 [−0.090, 0.120], *p* = 0.782). Other predictors such as the poverty index, age, device used and whether participants completed high school were shown to be influential to the effect. Table 6 demonstrates the results of the main analysis model. Figure 3 shows the relationship between experimental condition, poverty index and cognitive performance.

**Table 6.** Results of the main analysis.

term	estimate	std. error	statistic	*p*
(intercept)	0.79	0.07	11.12	<0.001
scarcity condition	−0.05	0.03	−1.85	0.064
poverty index	−0.10	0.02	−5.32	<0.001
scarcity condition × poverty index	0.01	0.03	0.28	0.782
age	−0.02	0.00	−21.37	<0.001
smart device	0.69	0.03	25.13	<0.001
uncompensated	0.05	0.05	0.99	0.320
highest education: high school	−0.10	0.04	−2.60	0.009

Note. Results from a linear regression analysis predicting the attentional performance index. Estimates represent standardised coefficients, except for age, which was not standardised. Standard errors are in parentheses. The scarcity condition and poverty index terms capture main effects, while their interaction term assesses SICI. Smart device, uncompensated and highest education: high school are dummy variables. The smart device variable distinguishes participants who used a tablet or smartphone from those who used a computer.

#### 3.2.1. Robustness analysis

**Figure 4.**
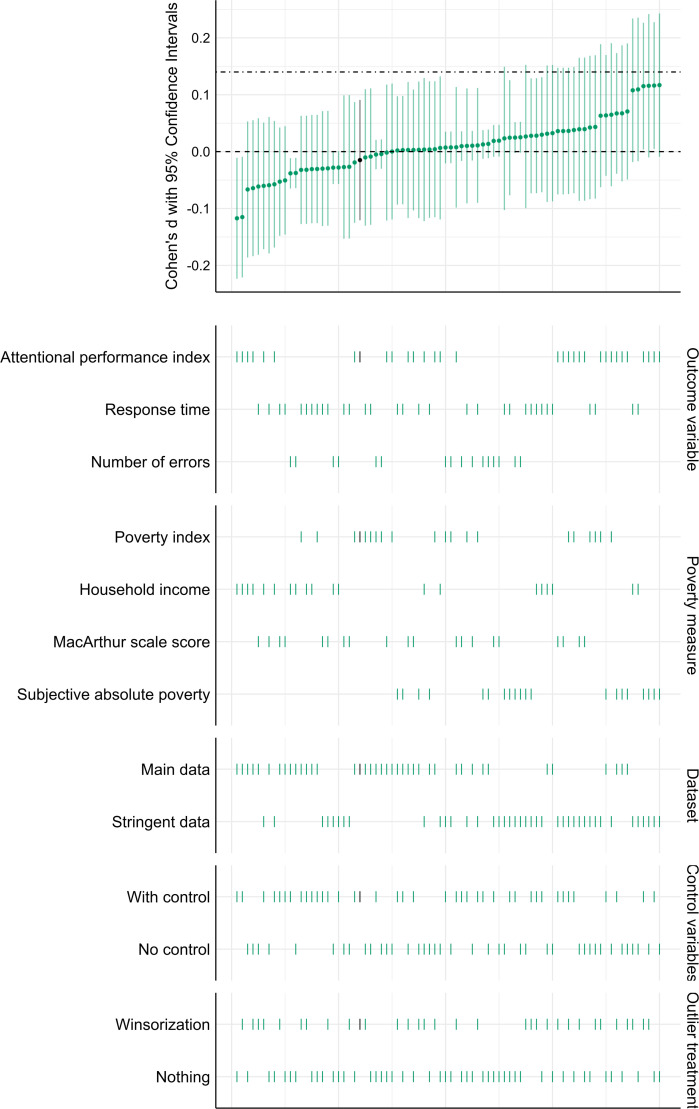
Descriptive specification curve depicting effect size estimates of the robustness analysis. The top panel displays 80 Cohen’s *d* effect sizes, each estimating the influence of the experimental manipulation on the impact of different financial status indicators on different attentional performance measures in the SART under different analytical specifications, with 95% confidence intervals shown. The dashed line indicates 0, while the dot-dash line indicates the effect size we can realistically detect based on the power analysis (*d* = 0.14). In the bottom panel, each row represents an analytical choice, with vertically aligned dots indicating observed estimates. The black dot in the top panel represents our primary analysis, and the black lines in the bottom panel represent the corresponding specifications.

Using the alternative, more stringent set of exclusion criteria, the final sample consisted of 3870 participants. The multiverse analysis supported the findings of the primary analysis. We found that 63 of the 79 performed additional analysis suggested that the effect is smaller than 0.14, 14 were inconclusive and 2 showed evidence for the existence of the effect. Figure 4 shows the distribution of the detected effect sizes. Electronic supplementary material, table S10 details the underlying data.

### 3.3. Exploratory analysis: generalizability of SICI

The following regression terms were consequently retained by the LASSO model: age (*P*_selection_ = 1), dummy of being retired (*P*_selection_ = 0.96), dummy of completing the test while travelling (*P*_selection_ = 0.87), if the participant was self-admittedly distracted (*P*_selection_ = 0.76) and if the participants had dependents (*P*_selection_ = 0.62).

#### 3.3.1. Robustness analysis

**Figure 5.**
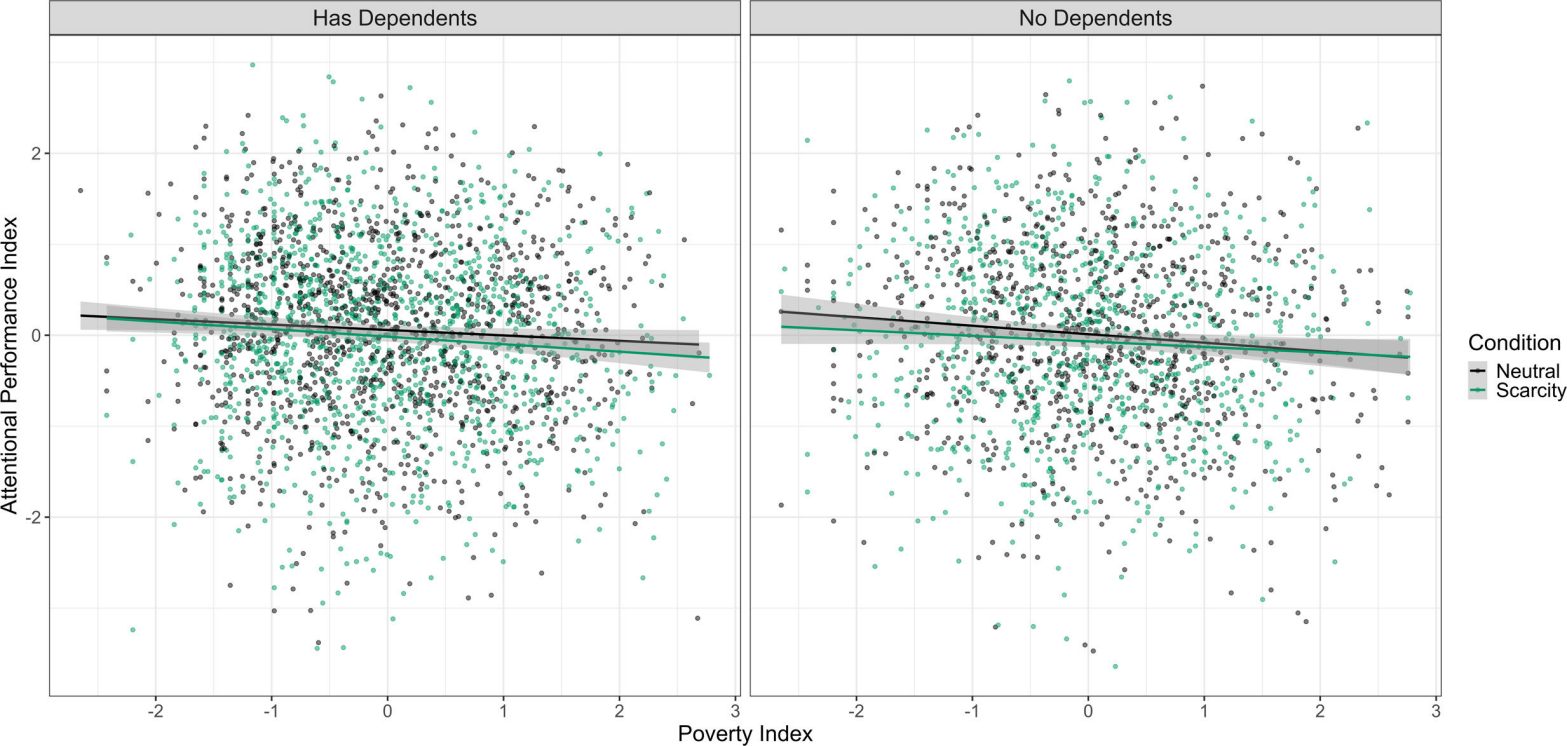
Scatter plot of poverty index and attentional performance index by experimental condition and having dependents. *Attentional Performance Index* and *Poverty Index* are mean-centred and standardized. The shaded grey areas around the regression lines represent 95% confidence intervals. *No Dependents* includes those who reported no income or no dependents.

After repeating the analysis using the alternative specifications, having dependents emerged as a moderating factor of SICI in one group of LASSO models. Figure 5 visualises the relationship between having dependents, poverty index, attentional performance index and experimental condition. Additionally, the poverty index and subjective poverty emerged as factors influencing performance in two and one groups of LASSO models, respectively. Electronic supplementary material, figure S3 visualizes and electronic supplementary material, table S11 details the results of the multiverse analysis.

### 3.4. Survey attrition

We found no evidence of differing survey attrition rates between the experimental and control conditions across any of the four main socioeconomic status measures: Poverty Index, Adjusted Household Income, Subjective Absolute Scarcity and MacArthur Scale Score. Electronic supplementary material, table S12 details these results.

### 3.5. Investigation of underlying mechanisms

#### 3.5.1. Financial worrying

We found no evidence that the stimuli in the scarcity condition triggered higher levels of financial worry than those in the control condition (OR = 0.970 [0.872, 1.081], *p* = 0.585), nor that they affected richer and poorer individuals differently (OR = 0.955 [0.853, 1.068], *p* = 0.418). All 15 models of the robustness analysis led to the same conclusions (electronic supplementary material, table S13).

#### 3.5.2. Relative- and absolute-scarcity-related thoughts

Cues in the experimental condition related to the shortage (absolute-scarcity) or abundance (relative-scarcity) of money influenced the overall prevalence of any type of scarcity-related thoughts during the SART task differently than neutral cues (OR = 2.596 [2.000, 3.405], *p* < 0.001). Moreover, this effect was observed for both absolute- (OR = 3.472 [2.339, 5.274], *p* < 0.001) and relative-scarcity-related cues (OR = 2.903 [1.938, 4.440], *p* ≤ 0.001). However, we found no evidence that absolute- and relative-scarcity-related cues differed in their impact on the prevalence of absolute-scarcity-related thoughts (OR = 0.737 [0.475, 1.114], *p* = 0.158) or on the prevalence of relative-scarcity-related thoughts (OR = 0.902 [0.613, 1.306], *p* = 0.591). All robustness tests (15 models across all three primary models) confirmed these findings. Detailed results are presented in electronic supplementary material, tables S14–S16.

### 3.6. Non-registered analyses

#### 3.6.1. Refining the primary analysis with LASSO-identified variables

Using insights from the primary exploratory analysis, we repeated the main analysis with different specifications to determine whether reducing noise in the data reveals the presence of SICI. First, we excluded participants who reported being distracted by environmental factors during the experiment, resulting in 3367 participants after applying the primary exclusion criteria and 3039 after applying the stringent criteria. Second, we incorporated the variables identified by the LASSO model: age, device used, compensation status and high school completion.

Before fitting the models, we tested whether socioeconomic status influenced the likelihood of reporting distractions in the two conditions. To do this, we replicated the analysis described in the *Attrition Analysis* section, with one modification: we excluded participants who reported being distracted. The results showed no evidence that socioeconomic status measures affected distraction reports differently across conditions. Details are presented in electronic supplementary material, table S17.

The analysis provided evidence against the presence of SICI (*β* = 0.029 [−0.030, 0.088], *d*_transformed_ = 0.058 [−0.060, 0.176], *p* = 0.334). Additionally, the robustness analysis reinforced this finding: only 2 of the 39 analyses supported the effect, 3 were inconclusive and the remaining 34 rejected its existence. Detailed results can be found in electronic supplementary material, table S18.

#### 3.6.2. Impact of dependents on scarcity-related thoughts and financial worries

Next, we tested whether the data support the impact of the three-way interaction—condition, socioeconomic status and having dependents, as identified by the LASSO regression—on the proposed mechanisms. We assumed that if the moderation effect exists, it might affect the supposed pathways of SICI: scarcity-related thoughts and financial worries. Accordingly, we fit two regression models, a binary logistic regression model with the scarcity-related thoughts outcome and an ordinal regression model with the financial worry dependent variable. Additionally, we repeated both analyses with both sets of regression criteria, all four poverty measures and with the inclusion of control variables that we used in the primary post hoc analysis. Learning from the results of the registered exploratory analysis, we only included those participants who reported not having been disturbed (*N*_primary_ = 3,367, *N*_stringent_ = 3039).

**Figure 6.**
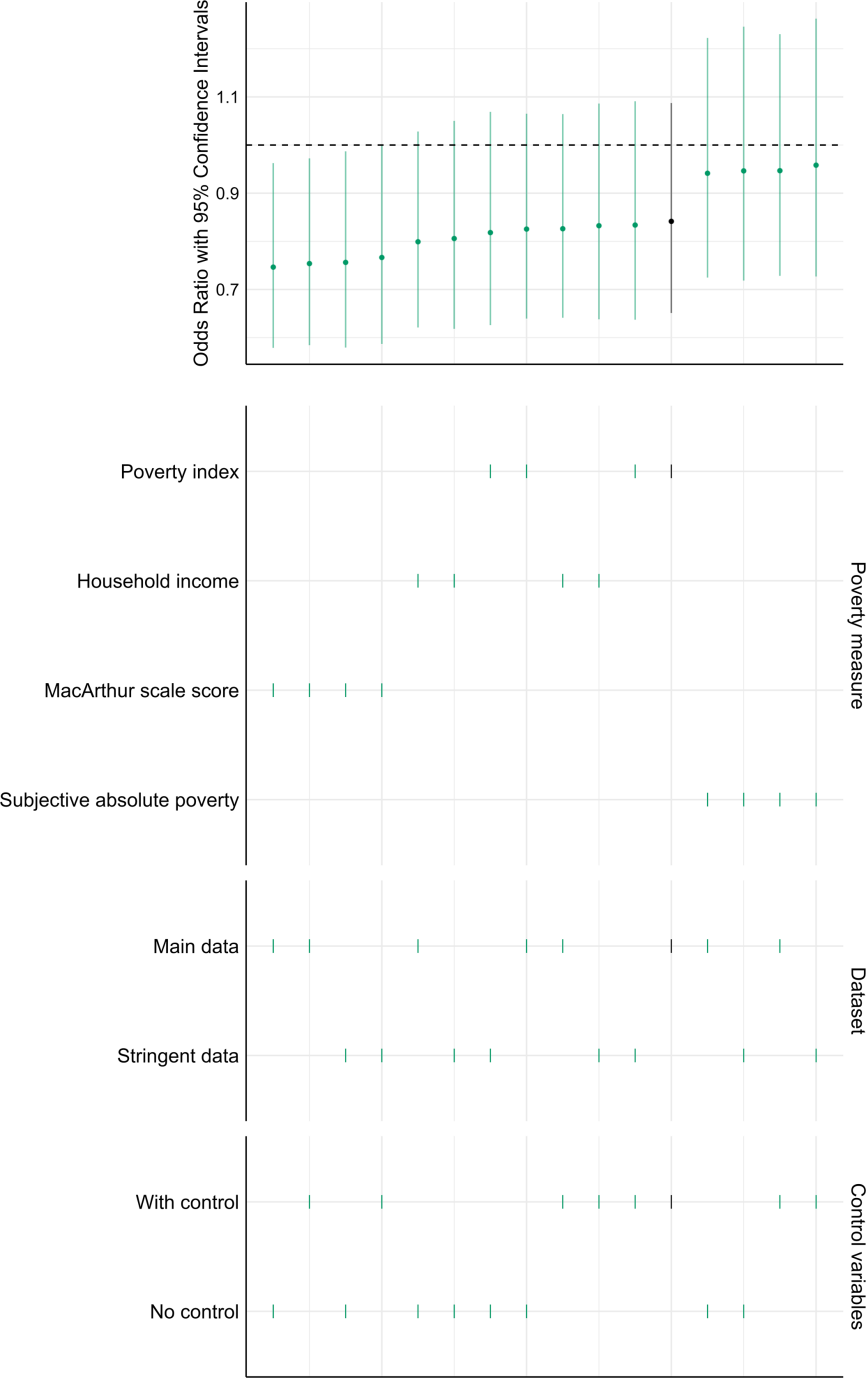
Descriptive specification curve depicting the three-way interaction of condition, socioeconomic status and having dependents on financial worrying. The top panel displays 16 odds ratios, each estimating the influence of having dependents on the impact of the experimental manipulation for the poorer and the richer on financial worrying under different analytical specifications, with 95% confidence intervals shown. The dashed line indicates 0. In the bottom panel, each row represents an analytical choice, with vertically aligned dots indicating observed estimates. The black dot in the top panel represents our primary analysis, and the black lines in the bottom panel represent the corresponding specifications.

In the case of the scarcity-related thoughts, the model predicting the presence of any type of scarcity-related thoughts (i.e. both absolute- and relative-scarcity) showed a non-significant result for the threefold interaction term (OR = 1.262 [0.722, 2.212], *p* = 0.415). This finding was supported by all models in the robustness check. Electronic supplementary material, table S19, details these results. Similarly, we found no evidence that financial worries are affected by this threefold interaction (OR = 0.841 [0.651, 1.087], *p* = 0.187). 12 of the 15 multiverse models supported this conclusion. Figure 6 visualizes and Electronic supplementary material, table S20 details the results of the multiverse analysis.

## 4. Discussion

In this article, we investigated whether the presence of financial scarcity-related cues disproportionately affects the sustained attentional performance of the poor in online contexts (SICI). Across an online sample of 4280 participants recruited in Hungary, we used an experimental design in which participants completed a word association task featuring absolute- and relative-scarcity-related, or neutral cues, followed by a Sustained Attention to Response Task (SART). The analysis revealed no significant interaction between the socioeconomic status composite index derived from different dimensions of scarcity and condition, with the estimated effect size not only statistically non-significant (*d* = 0.015, *p* = 0.782) but also significantly smaller than our predefined smallest effect size of interest (*d* = 0.14). This provides evidence against the existence of SICI under the tested conditions and suggests that if it exists, the effect is small. This conclusion was robust against various alternative analytical specifications. To determine whether unexplained variance may have masked the presence of SICI, we conducted further non-registered analyses aimed at reducing noise using insights gained from our exploratory analyses. This investigation provided further evidence against the existence of a universal SICI. These negative findings align with the meta-analysis by Szecsi & Szaszi [24] but contradict some prior published results [2,4,16].

Despite our efforts, examining the heterogeneity of the effect utilising LASSO regression analysis with the inclusion of over 400 predictors (including two- and threefold interactions) only led to one potential revelation: having dependents may increase the prevalence of SICI. However, this finding should be interpreted with caution, as only one of the 10 LASSO model sets selected this term. Post hoc analyses failed to support that, in the presence of financial-related stimuli, either financial worrying or scarcity-related thoughts increased more for the poor than for the rich when they had dependents. Future research on SICI should explicitly examine whether having dependents significantly affects its prevalence.

Our inability to detect universal SICI may be due to the properties of the stimuli we used, as our study diverged from the existing literature in three key ways regarding the scarcity-related cues, we employed.

First, of the two pathways discussed in the literature, financial worries and scarcity-related thoughts, we found evidence that only the latter was influenced by our experimental manipulation. Notably, relative-scarcity-related (abundance-related) cues increased the prevalence of these thoughts, similarly to absolute-scarcity-related cues. Given that we found no evidence for SICI in our data, this suggests that if SICI exists, it may not operate through the frequency of scarcity-related thoughts. Alternatively, it is possible that while the used stimuli increased the prevalence of these thoughts, its effect was not large enough to translate into affecting attentional performance. Future studies should aim to identify and utilize cues that specifically impact financial worrying to test whether this pathway underlies SICI.

Second, unlike earlier studies (e.g. [2,16]), we used stimuli that were linked to the general concept of absolute and relative scarcity but not directly connected to participants' personal financial struggles. Using cues that reflect real-life financial problems could evoke stronger emotional responses and memories, potentially disrupting attentional performance to a greater extent. One possible approach to overcome this limitation could be a two-step design used in the mind-wandering literature [13]: in the first step, participants are asked to describe their financial concerns, while in a later step, the experimental stimuli are tailored to the specific type of worries expressed by the returning participants.

Third, our stimuli did not create a ‘story’ or situational context as earlier designs, such as maths questions and hypothetical scenarios, did. Our results suggest that merely presenting scarcity-related words may not be sufficient to evoke an experience of scarcity or a strong emotional response; participants may need to feel immersed in a scarcity-related context conveyed by the cue. Consequently, our findings provide evidence against a universally generalizable SICI.

Recruitment methods may have also contributed to our null findings. Although we specifically gathered data from economically disadvantaged and less educated participants, because participation was voluntary, those whose cognitive capacities were more heavily restricted by scarcity may have been less likely to volunteer. As a result, our sample may have underrepresented individuals most affected by SICI. Overcoming this limitation would likely require natural experiments, such as those conducted by Duquennois [4].

## 5. Conclusion

To summarize, our findings challenge the generalizability of scarcity theory and the existence of SICI. Specifically, we provide evidence against the prediction that textual scarcity cues universally impair cognitive performance. It is possible that having dependents could enhance the effect. The generalizability of our results is restricted by the properties of the cues we utilized. Additionally, our findings may not extend to individuals in deep poverty or those facing such severe financial strain that they would not pause their daily struggles to participate in an online experiment.

The contrast between recent positive findings [2,4] and negative findings [18,20], including the high-powered null results presented in this article, suggests that scarcity theory fails to predict behaviour across different contexts and requires refinement. In particular, the conflicting results of Duquennois [4] and the present study highlight the need to identify the mechanisms linking scarcity-related cues to cognitive tunnelling or increased cognitive load. Understanding which cognitive processes were activated in the children studied by Duquennois [4] but not in our sample may help specify the conditions under which SICI emerges. Based on our findings, one plausible explanation is that financial worrying is the key mechanism. The methods developed here can guide future research on these mechanisms in SICI. However, to determine which mechanisms connect cues to cognitive outcomes, future studies must use stimuli that target all relevant pathways, such as financial worrying. These stimuli must be developed with ecological validity and remain grounded in the lived experiences of those facing poverty.

## Data Availability

The Supplementary Materials are available at https://osf.io/ngtzw. The analysis code and data are available on the OSF page of the project at [67]. The source code of the utilised SART and instructions for running it are available on GitHub at [68]. Supplementary material is available online [69].

## Footnotes

^1^
Previous research has used the terms ‘scarcity theory’ (e.g. [5,6]), ‘psychological responses to scarcity’ [7], and ‘resource scarcity’ [8] partially to describe the impact of scarcity on cognitive functioning, but also to explain other related phenomena such as trade-off thinking and borrowing behaviour [9]. However, in this article, we specifically focus on the predictions related to cognitive performance.

^2^ Note, that although SICI shares similarities with the extensively studied concept of money priming, there are important differences. First, the money priming literature examines the direct presence of money (picture, physical touching, words describing money, etc.), while here, we focus on the salience of broader concepts associated with finances (e.g. a bill or a Rolex watch); second, money priming research focuses on the main effect of money priming, while here we focus on how the effect disproportionately impacts different income groups. For an extensive review of money priming, see Lodder *et al.* [25].

^3^ In a linear regression model that includes one predictor.

^4]^ This approach relies on word embeddings, which are vectorial representations of words based on their proximity and interchangeability in text. Prior research [39] has shown that word embeddings from LLMs and human judgements partially overlap, making this method effective for stimulus selection. We used the ‘text-davinci-003’ GPT 3.5 model [40], as it is one of the most advanced language models that were accessible and tuneable at the time of the preparation of the stimuli.

^5^ We could not apply the same strategy for the creation of neutral stimuli as for the scarcity salience, because our approach is based on the identification of words and expressions close to scarcity in the underlying word embedding (i.e. words with the highest cosine similarities) and we have no knowledge of a prompting method that would identify words and expression in the opposite end of the multidimensional space of the word embedding (i.e. words with the lowest cosine similarities).

^6^ Helmert coding is a type of contrast where the second level is compared to the first one, the third level to the mean of the first two, the fourth to the first three and so on. It is suitable for ordinal variables where it is difficult to determine the differences between which two levels are influential.

^7^
In the accepted stage 1 registration, we committed to first finish the recruitment through news sites, and only then start recruitment through Facebook. In practice, we started recruiting participants through Facebook before, and continued to do so during the time news outlets published our call for participation. This was an oversight; we anticipated needing additional participants and overlooked the fact that we had registered the recruitment order.

^8^
Compared to the accepted stage 1 protocol, we edited this sentence for clarity. We added the words ‘While testing the main hypothesis,’ because for other effects, a reliable estimate of the smallest interesting effect size was not available, preventing the use of equivalence testing.

^9^
In the accepted stage 1 protocol, we committed to the use of a dummy variable indicating if participants completed high school or not. We changed it due to the small number of participants (112) who had not completed high school. The original categorization was impractical for analysis as it led to highly uneven sample sizes.

^10^
In the accepted stage 1 protocol, we committed to the inclusion of replies where the associations were meaningful words in the Hungarian language. During the process, we realized that the original criterion would exclude participants who appeared to be genuinely engaged, as we identified meaningful replies that were not part of the Hungarian language (e.g. English words).
